# Supplementary material for: Are carbon emissions trading and green financial instruments synergistic? -Comprehensive quantitative research based on content analysis
Source: PLoS One. 2024 Mar 7;19(3):e0298601. doi: 10.1371/journal.pone.0298601 (PMC10919601; doi:10.1371/journal.pone.0298601)
Supplement: S1 Data — (ZIP) [file pone.0298601.s001.zip › Data/地级市Z_F工作报告.zip!/╔┬╬≈/╔╠┬σ╩╨/└┤╫╘╣1⁄2╓┌║┼í╛┬φ┐╦╩2╛▌═°í┐_2013.docx]

各位代表：

现在，我代表市人民政府向大会作工作报告，请予审议，并请市政协委员和其他列席人员提出意见。

一、2012年政府工作回顾

2012年，面对经济下行压力加大的不利局面，全市上下坚持以邓小平理论、“三个代表”重要思想、科学发展观为指导，牢牢把握科学发展、富民强市主题和发展循环经济主线，确定了稳为基础、进为目标的工作思路，认真落实中省一系列预调微调措施，克难攻坚、埋头苦干，全面完成了各项目标任务。全年实现生产总值439亿元，增长14.8%；财政总收入33.7亿元，增长26.1%，地方财政收入21.5亿元，增长30.3%，财政总支出134亿元，增长25.5%;城镇居民人均可支配收入19998元，农民人均纯收入5425元，分别增长15.3%和18.3%；城镇登记失业率控制在2.96%；全年物价上涨2.3%；万元GDP能耗下降3.38%，二氧化硫、化学需氧量、氨氮和氮氧化物排放分别削减2.3%、2.5%、1.7%和0.1%。

(一)着力调结构、转方式，产业转型升级迈出新步伐。把循环发展作为经济结构调整的主方向，市财政安排循环经济和产业发展专项资金6176万元，争取中省循环经济项目资金1.7亿元，实施循环经济和尾矿资源综合利用项目42个，其中铅锌尾矿生产建筑用砖等8个项目建成投产，商洛列入国家第一批尾矿资源综合利用示范基地。采取减免企业税费、收储大宗工业产品等十项措施促销保产稳市场，有效缓解了企业的生产经营压力。通过财政列支和专项资金扶持等措施鼓励企业增加投资，争取中省专项资金8900万元，市财政投入工业支持资金1000万元，新增规模以上工业企业35家，规模以上工业增加值108.9亿元，增长34.2%。出台了加快服务业发展的若干政策意见，扶持发展旅游、物流、商贸等产业，积极扩大消费需求，消费潜力进一步释放，社会消费品零售总额107.3亿元，增长16.4%。全市投入1.2亿元开展旅游宣传促销，成功举办第三届中国秦岭生态旅游节，天竺山、牛背梁创建4A级景区通过国家旅游局正式命名，牛背梁加入世界“人与生物圈”保护区，金丝峡国家森林公园荣获全国生态旅游创新奖，全市接待游客2288万人次，实现综合收入102.5亿元，分别增长34.3%和50.4%。出台了进一步加快农业产业化发展的若干意见，发展无公害农产品基地311万亩，我市烟叶生产首次跨入全国重点产区，全市建成省级现代农业示范园区8个，培育省级以上龙头企业22家，朝阳工贸公司成为全市首家国家级龙头企业，商洛被省政府命名为全省核桃产业强市。农民专业合作社发展到876家，辐射带动5.8万农户。粮食总产64.3万吨，蔬菜总产65万吨，肉、蛋、奶产量分别达到24万吨、7万吨和1900吨。商洛农产品创建陕西省著名商标6个，“金丝源”品牌获得国家驰名商标，山阳被省政府授予陕西省质量兴省先进县。

(二)着力上项目、增投资，经济社会发展形成新支撑。以项目为抓手扩大固定资产投资，实施的150个市级重点项目完成投资231亿元，全社会固定资产投资391.6亿元，增长27%。以循环项目和一县一产业项目为重点，实施重大产业项目99个，延长氟化工系列产品开发等9个项目建成投产。筹措项目前期费1.44亿元，大西沟铁矿开发等85个项目前期工作进展顺利。加大基础设施投资力度，宁西铁路复线征迁工作全面推进，西商二线建成通车，全市高速公路里程突破400公里，实现了县县通高速的目标。改造县乡公路160公里，新修通村水泥路200公里。110千伏西康铁路复线供电、罗庄输变电等工程竣工投运，改造升级10千伏及以下农村电网线路1462公里。气象塔楼和多普勒天气雷达建成投用。实施土地整治3.1万亩，建设基本农田9.17万亩，保障发展用地2.09万亩，新修加固堤防251.8公里，新建农村饮水工程164处，解决饮水困难27.7万人。完成造林绿化34.1万亩，治理水土流失810平方公里，丹凤“桃花谷”建成全省首个水保科技示范园。

(三)着力抓管理、促建设，城镇基础条件发生新变化。商洛市第二轮城市总体规划通过省政府审批。组织开展“城市建设管理年”活动，启动实施各类城建工程151个。中心城市基础设施建设完成投资10.6亿元，丹南新区路网一期工程实现路通灯明，西商高速城区连接线和龟山大道建成通车，环城北路控制性工程全线建设，西街旧城改造拆迁工作基本完成，商鞅大道东段主体工程基本完工，西商高速城区连接线跨丹江大桥主体完工，312国道商丹段“二改一”控制性工程启动实施。出台了推进重点镇建设的八条政策措施，沙河子省级重点示范镇及17个省、市重点镇加快建设。全市完成危房改造1.6万户，农村居民进城落户8.13万人，全市城镇化率提高了2.7个百分点。出台了中心城区违法建设查处暂行办法，集中查处了一批违法建筑。六县县城建设快速推进，污水、垃圾处理厂建成运营。丹凤、山阳两县创建省级园林县城通过验收，丹凤创建省级卫生县城挂牌命名，山阳创建省级卫生县城通过省爱卫会考核验收。

(四)着力惠民生、强保障，群众幸福指数有了新提高。新增财力用于民生的比重达到82%，实施的1045个基本建设类民生项目已经建成1025个。深入推进扶贫开发，9.1万贫困人口脱贫。扎实推进移民搬迁工程，2011年搬迁任务超额完成，在省政府考核中获得一等奖。2012年新建集中安置点201个，搬迁开工2.1万户，房屋主体完工1.7万户。加大保障性住房建设力度，全年完成投资38亿元，新开工1.39万套，累计竣工4.1万套，交付使用3.6万套。千方百计促进就业，发放小额担保贷款7.1亿元，新增城镇就业1.44万人，农村劳动力转移就业66.7万人。七县区全部纳入城乡居民养老保险国家试点县，143万城乡居民参保，29.6万居民享受待遇。新农合筹资和城乡低保补助标准持续提高，残疾、高龄、特困等群体待遇保障有效落实。新建、改扩建中小学校80所，公办幼儿园30所，市幼儿园扩建工程启动建设，市特殊教育学校建成投用。山阳、商南两县“双高双普”创建通过省政府命名表彰，山阳创建教育强县通过省政府前期评估验收。各级各类教育加快发展，高考二本以上上线率连续五年全省第一。完成了36个镇办文化资源共享工程，启动了广播电视“户户通”工程。举办了国际青年男篮四国挑战赛和环中国国际公路商洛段自行车赛，我市体育健儿在第十四届伦敦残奥会和省十五届运动会年度比赛中获得11金7银的好成绩。县级公立医院综合改革取得阶段性成效，新农合参合率连续九年全省第一。人口和计划生育工作持续加强，出生人口政策符合率达到98.2%。

(五)着力促创新、增活力，深化改革开放实现新突破。全年投入各类科技创新资金3.1亿元，鼓励科研机构、科技人员与实体经济结合，科技创新平台建设取得新进展，院士工作站正式落户商洛，市材料科学工程院投入运行，建成企业技术研发中心15个。与26家高校和科研单位开展科技合作，组织实施重大科技专项8个，全年专利申请量561件，技术合同交易额3030万元。完成了工商、质监体制调整。加快推进财税各项改革，国库集中支付改革实现市县区全覆盖。加强行政事业单位经营性资产管理，建立了国有资本经营预算制度。出台了金融支持实体经济等六个指导性意见，小额贷款公司发展到10家，长安银行商洛分行正式营业，陕西炼石实现借壳上市，林权抵押贷款深入推进，农村房屋抵押贷款试点进展顺利。开展了优化发展环境专项整治活动，出台了招商引资考核和奖励办法，全年招商引资到位资金227亿元，增长53%；利用外资8461万美元，增长70.9%。非公有制经济增加值219.6亿元，占到全市生产总值的50.03%。

(六)着力改作风、提效能，创新社会管理取得新成效。完善抓落实工作机制，推行“月考季评”制度，政府的执行力进一步提升。积极推进社区管理规范化、专业化，改扩建城市和农村社区服务站81个。深入开展“三问三解”活动，健全完善了信访工作机制，及时排查调处了一批信访积案，商洛信访工作经验得到中联办的肯定和推广。扎实推进平安商洛建设，圆满完成“853”工程，应急处置能力明显提高，全市安全生产形势总体平稳，社会治安满意率、平安创建知晓率和公众安全感综合指数均位居全省第一。加强依法行政能力建设，办理人大代表建议174件、政协委员提案307件、议案34件。扎实推进政务公开和廉政建设，加强领导干部经济责任审计，积极推行预决算公开，严格控制公费出国出境，政府的公信力不断提升。

过去的一年，我市其他方面工作也取得了明显进步。统计、工商、药监、质监、人防、水文、方志、档案、外事、侨务、民族、宗教等工作取得新成绩，慈善、通信、无线电、工商联、妇女儿童、红十字会、国防动员等各项事业有了新发展。

各位代表，过去的一年，我们在复杂严峻的形势下，取得了令人鼓舞的成绩。这些成绩来之不易，是省委、省政府和市委正确领导的结果，是市人大、市政协监督支持的结果，是全市人民实干苦干的结果。在此，我代表市人民政府，向全市广大干部群众和离退休老同志，向驻商部队指战员、武警官兵和公安民警，向参与商洛建设的投资者和企业家，向支持政府工作的各位代表、各位委员和各界人士，表示崇高的敬意和衷心的感谢！

在看到成绩的同时，我们也清醒地认识到，我市经济社会发展仍面临许多矛盾和问题。主要是：自主创新能力不强，产业层次较低，转型升级任务艰巨；能耗水平依然偏高，环境总量指标不足，建设用地供需矛盾突出；城市配套设施不够完善，市民的城市意识有待提高，城市管理水平亟待提升；体制机制不相适应，市场主体不足不优，开放水平还需提高；社会管理存在薄弱环节，人民内部矛盾多样多发，创新社会管理任务繁重；少数干部的思想观念不够解放，政府工作中的官僚主义和奢侈浪费现象不同程度存在，廉政勤政建设和反腐败斗争仍然是长期任务。对于这些问题，我们将采取措施，切实加以解决。

二、2013年主要工作任务

今年是我市全面实施“十二五”规划承前启后的关键一年。我们要深入贯彻落实党的十八大精神，按照“五位一体”的总体布局，紧紧围绕科学发展、富民强市主题和发展循环经济主线，将稳增长放在更加重要的位置，更加注重转型升级，更加注重科技创新，更加注重城乡统筹，更加注重改革开放，更加注重民生改善，更加注重社会和谐，巩固和发展全市经济社会发展的良好势头。今年的主要预期目标是：生产总值增长13.5%左右，财政总收入和地方财政收入均增长17%，全市居民人均可支配收入增长15%以上，其中，城市增长15%左右，农村增长16%左右，城镇登记失业率控制在4.5%以内，物价涨幅控制在4%以内，节能和减碳指标控制在省上确定的范围以内，二氧化硫、化学需氧量、氨氮和氮氧化物排放分别削减2%、2%、2%和3%。

(一)实施项目带动战略，培育新的经济增长点

扭住项目建设不动摇，抓住招商引资不放松，以招商抓项目，以项目增投资，确保全年固定资产投资增长26%。

一是加大招商引资力度。实施千名招商人才培养工程，健全完善市、县区两级招商联动机制，坚持引资、引技、引智相结合，有针对性地赴珠三角、长三角、环渤海等地区招商引资，扎实推进央企进商、民企进商、港澳招商和十七洽招商等签约项目的跟踪落实，更加积极主动地承接产业转移，力争全年招商引资到位资金290亿元。

二是狠抓重点项目建设。实施市级重点项目150个，年度投资255亿元以上。强力推进100个重点产业项目，突出抓好商南柏伦国际包装产业园、镇安抽水蓄能电站等项目建设，力促山阳天之润中药中间体提取等20个项目建成投产。积极配合西康、宁西铁路复线建设，抓好榆商高速和山柞高速前期工作。做好312国道丹凤至商南段和203省道市区过境公路升级改造前期工作，完成100公里县乡公路改造和180公里通村水泥路建设任务。加快市一级客运站和城镇站点配套建设，启动商南、丹凤两个二级客运站建设，镇安二级客运站建成投运。实施1个330KV、2个110KV、9个35KV输变电工程，继续抓好城网农网改造升级工程。加快推进无线城市建设，新建城乡通信基站460个。

三是加强项目前期工作。筹措前期费1亿元以上，扎实推进150个重大项目前期工作。认真研究国家产业政策和投资导向，积极策划装备制造、新能源、新材料、综合利用、农产品深加工、仓储物流、休闲养老、文化旅游等产业项目，力争策划一批投资过50亿元、过100亿元的战略性项目，突出做好火电厂、西安(商洛)机场、石煤综合利用、钾长石深加工、石墨精深加工、矿钢一体化、特种汽车、大秦岭秀等重大产业项目前期工作，确保市级储备库项目达到1000个以上。

四是强化项目建设保障。大力实施土地综合整治、水毁耕地修复、“空壳村”治理、工矿废弃地复垦和建设用地增减挂钩等项目，严格落实闲置土地处置办法，加快“退二进三”步伐，依法清理低效利用和供而未用土地，增强项目用地保障能力。采取BT融资、基金融资、土地拍卖、融资租赁等方式拓宽融资渠道，鼓励和支持县区成立城投公司，市城投公司发行城投债15亿元。继续深化政银企合作，年内新增贷款40亿元以上，完成各类保费收入10亿元。支持盘龙制药等重点企业做好上市前期工作。

(二)推动产业结构调整，加快提升产业竞争力

坚持一产强基础、二产扩规模、三产促突破，加快产业结构战略性调整，构建具有商洛特色的现代产业体系，实现有质量、有效益、可持续发展。

一是推进工业经济转型升级。运用现代信息技术和强化科技创新，推进矿产、医药、食品、建材等传统产业改造升级。制定支持现代材料工业基地建设的政策措施，积极推进矿产资源整合及深度开发，加快发展现代材料产业。抓住中省扩大光伏产品市场应用机遇，支持比亚迪300兆瓦太阳能电池组件项目建设，大力推进太阳能光伏产业发展。加快推进大功率LED、新工艺保温板、建筑机械等生产线建设，不断培育壮大节能环保、机械制造等产业。加强核心关键技术攻关，促进科技成果产业化，加快发展新能源、生物技术等新兴产业。继续支持商丹园区创新发展，抓好县域工业集中区建设，全年新增入园企业40户。以“抓销售、稳生产、保市场”为重点，加强工业运行监测分析，支持企业加大产品促销，加强企业与金融机构对接，加快推进40个重点工业项目建设，力争主要工业品产销率达到90%以上，规模以上工业增加值增长25%以上。

二是推进现代农业做优做强。认真贯彻落实中央《关于加快发展现代农业进一步增强农村发展活力的若干意见》，创新农业生产经营体制，健全农业社会化服务体系，提高农业产业化发展水平。大力调整种养结构，加快土地流转，加强科技推广，提高粮食生产、“菜篮子”工程和其它多种经营的综合效益，确保粮食总产55万吨左右，蔬菜总产70万吨。继续推进核桃、板栗、茶叶等产业整合，全年培育发展市级以上农业产业化龙头企业10个。严格执行农产品质量安全标准，扎实做好“三品一标”认证工作。加大全国驰名商标、省级著名商标培育力度，提高商洛农产品的知名度和市场竞争力。继续加大现代农业示范园区建设力度，新建市级以上现代农业示范园区10个，其中省级园区3个以上。启动实施“职业农民塑造工程”，加快培养新型职业农民队伍。加强农田水利建设，新建基本农田7万亩，新修加固河堤100公里，新建农村饮水工程100处，完成11座小型病险水库除险加固。加强农业气象服务，加快水文监测体系建设。扎实推进新农村建设，实施扶贫整村推进项目115个，创建产业扶贫示范村30个，力争8万贫困人口实现脱贫。

三是推进服务业提档升级。继续做好“秦岭最美是商洛”品牌提升，坚持用园区的理念建设旅游景区，推进旅游与休闲农业、商贸、餐饮、文化等产业有机融合，加快丹凤棣花文化旅游区、商南金丝峡旅游示范区、柞水营盘生态旅游产业园建设。加强景区周边环境和旅游秩序治理，加大旅游景点建设和创建力度，完成商洛旅游标准化示范城市创建任务，推进柞水省级旅游示范县创建工作，力争山阳月亮洞、商南闯王寨等景点在“十一”前开放，启动柞水溶洞和丹凤丹江漂流4A级景区创建。支持丹凤挖掘人文资源，促进旅游错位发展。加强在中省主流媒体和周边城市的旅游促销，筹办好第四届中国秦岭生态旅游节和陕川甘旅游年会，力争全年接待游客突破2600万人次。编制完成西安第二生活区建设规划，大力发展度假、养老、疗养等生活性服务业，积极发展物流配送、电子商务等新型业态。落实好国家房地产调控措施，引导房地产市场规范健康发展。

四是推进城乡消费快速增长。继续增加财政用于改善和扩大社会消费的支出比重，落实流通领域税收减免和信贷刺激消费的有关政策，严格执行带薪年休假制度。扶持文化、休闲、体育和健身消费，发展住房、汽车消费，培育网络和信用消费，完善建材企业与保障房、重点镇、移民搬迁等工程的对接机制。继续实施万村千乡市场工程和新农村现代流通网络工程，搞好农超对接、农产品冷链物流和蔬菜进社区等工作，加快消费基础设施建设，加强消费市场监管，确保城乡居民基本生活品有效供给、价格稳定和消费安全，全年社会消费品零售总额增长16%。

(三)加快推进科技创新，增强经济发展的内生动力

坚持创新驱动、人才引领，继续实施“51158”科技带动工程，提高全市科技创新水平，激发经济增长的新动力。

一是增强企业创新能力。进一步健全完善推动科技创新的政策措施，市财政继续安排1000万元以上资金支持科技创新工作。强化企业科技创新主体地位，引导企业增加科研投入，年内新建企业研发中心10个。加快科技成果转化应用与推广，围绕主导产业实施10个重大科技专项，年内实施专利技术孵化项目10个，完成技术合同交易额3500万元。

二是完善科技创新平台。强化协同创新、共同发展理念，坚持政府推动与市场配置相结合，促进科技创新平台发挥作用。整合商丹园区院士工作站、市材料科学工程院等五大创新平台，组建钒、钼研发中心及配套中试中心，启动尾矿资源综合利用重点实验室中试及检测，支持比亚迪新能源研究所开展原材料及危废品检测评估，推进中药材GAP科研工程中心开展桔梗指纹图谱研究，加快核桃研究所科研楼建设并投入运营。

三是培育科技创新人才。制定人才培养引进计划，开展新一轮“十百千”人才评选工作，积极引进各类高端人才、领军人才和创新创业团队，重视培养本土人才和各类实用人才。继续完善科技特派员制度，大力推广首席工程师制度，支持企校对接开展订单式培训，增强大学生创新意识和实践能力。完善人才使用管理激励机制，对有突出贡献的优秀人才实施重奖。

(四)加强城市建设管理，提升城镇的承载能力

以建设“最美城市”为目标，推进“城市建设管理年”活动，加快推进新型城镇化进程，全市城镇化率达到45%以上。

一是精心规划城市。进一步强化规划意识，创新规划理念，加大规划投入，以规划指导建设，用规划促进管理。实行规划公示制度，强化规划执行管理，对一些重点区域和重大建设项目的规划设计，采取方案征集、招投标等方式确定，增强规划的科学性、公开性和权威性。完成商丹一体化总体战略规划、杨峪河片区和刘湾片区控制性规划，做好城周马莲峪等6条沟峪规划编制以及环城南路城市设计，启动中心城市地下空间规划设计，抓好城市“绿道”规划和市区重要区域、主要街路的城市景观设计，做好六县县城总体规划的编制和审批工作，编制完成17个省、市重点镇的建设规划。

二是精美建设城市。按照城镇建设投资占固定资产投资10%以上的要求，全年完成城镇建设投资50亿元。加快中心城市基础设施建设，完成投资20亿元以上，环城北路年底前竣工通车，商鞅大道东段6月底前建成，西街旧城改造完成安置房主体工程，莲湖东侧旧城改造5月底前完成征迁任务并启动安置房建设。加快推进城中村改造，着力改善城市居民的居住条件。扎实推进商丹一体化建设，加快312国道商丹段“二改一”工程进度，积极做好丹凤撤县设区前期工作。加快六县县城基础设施建设，提高县城建设和管理水平。积极推进商州-丹凤、镇安永乐镇-云盖寺等6个城乡统筹示范区建设，抓好沙河子省级重点示范镇新区中医院、文体中心等工程建设，完善供水、供电、燃气管网等基础设施。加大17个省、市重点镇建设力度，把重点镇建设、移民搬迁、小村并大村、农村居民进城落户结合起来，引导人口合理布局和有序转移。

三是精细管理城市。坚持建管并重，夯实四级责任，加强分区分段监管，推进城市管理“网格化”。整合信息资源，建设数字城管，实现管理对象和作业标准“数据化”。加大城市硬化、亮化、净化和美化工程建设力度，突出解决交通拥堵、市容不整和占道经营等问题，推进城市环境“靓丽化”。加强城管队伍建设，牢固树立是非观念，加大城管执法力度，严肃查处各类违法建设行为，推进城管执法“规范化”。启动中心城市创建国家园林城市工作，制定并实施城区及城周荒山绿化三年规划，实现城市建设“园林化”。

(五)全面深化改革开放，增添经济社会发展活力

坚定不移地深化各项改革，更加积极主动地扩大开放，以改革促开放，以开放促发展。

一是深入推进各项改革。启动收入分配制度改革，逐步缩小收入分配差距。继续推进财政体制改革，增强县区政府基本公共服务保障能力，建立镇办政权运转经费管理机制。加快推进金融体制改革，支持镇安农合行和柞水、商南农信社向农村商业银行改制。深入推进事业单位分类改革，积极推进文化体制改革，深化县级公立医院综合改革。继续深化国有企业改革，完善各类国有资本管理体制。推进供销社等行业改革，继续深化农村综合改革。

二是全面提升开放水平。主动加强与关天经济区城市的合作，积极增进与中西部经济技术协作区的合作，进一步加强与南京对口友好城市的经贸合作，广泛开展与国外友好城市的交流，提升开放型经济发展水平。用足用活外贸扶持政策，支持太阳能电池、有色金属和农副产品扩大出口，大力发展服务外包和服务贸易，促进外贸持续快速增长，实现进出口总额7600万美元。

三是发展壮大市场主体。认真落实国家“新36条”及42个实施细则，支持民间资本进入交通、水利、教育等领域，鼓励支持全民创业和民营业主二次创业，年内新增私营企业400户，新增个体经营户5000户，非公有制经济增加值占GDP的比重保持50%以上。强化“小进规”目标考核，确保新增规模以上工业企业25家。研究制定支持小微企业发展的政策措施，加快中小企业孵化园建设，促进小微企业健康快速发展。

(六)加强生态文明建设，增强可持续发展能力

把绿色生态作为品牌来打造，把循环利用贯穿于各个领域，大力实施生态文明建设，不断提升经济发展质量。

一是坚定不移地推进循环发展。积极创建国家级循环经济示范城市，以产品上下游衔接和资源综合利用为重点，在钢铁、水泥、化工等重点行业，推广清洁生产和循环利用技术，继续推进“三废”和尾矿综合利用，实施好39个循环项目和14个尾矿综合利用项目。加快推进循环经济园区建设，深入开展循环型企业创建，着力培育五洲钒矿等5户“双百工程”循环骨干企业。

二是更加有力地推进节能减排。加强新项目节能评估和环境影响评价，健全节能减排指标监测和考核体系，完善生态补偿和排污权交易制度，加快建立节能减排倒逼机制，坚决淘汰改造高耗能、高污染企业。严格控制主要污染物排放，加强机动车氮氧化物控制，搞好重点非电行业脱硫脱硝，推进城镇垃圾分类无害化处理。全面推行工业、建筑、交通和公共机构节能，重点抓好30家耗能大户的节能降耗。严格执行土地用途管制制度，加大耕地保护力度，节约集约利用土地。实施最严格的水资源管理，积极推广中水利用。

三是扎实有效地推进生态治理。加快推进陕西东南大门造林绿化，继续实施林业重点工程，巩固扩大退耕还林成果，全年造林绿化31万亩，不断提高森林覆盖率。加强丹江湿地保护，加快丹江流域综合治理，完成“丹治”二期17条小流域治理任务，治理水土流失800平方公里。抓好矿山开发区域生态恢复治理，进一步优化国土开发空间。完成中心城市省级环保模范城市创建任务，启动商州、镇安、丹凤三县农村环境连片整治工作，确保商南、镇安、柞水省级生态县创建通过验收。

(七)大力优化发展环境，打造加快发展的新优势

把优化发展环境作为把握机遇的实际措施来推进，下大气力建好硬环境、提升软实力，以环境优势催生发展优势。

一是创造吸引投资的基础环境。加强工业园区水电路视讯等基础设施建设，加快完善园区商贸流通、公用事业、金融网点和服务平台等配套设施，进一步增强和优化园区承载功能，使更多企业和资本向园区汇集。统筹考虑城市空间、产业、资源、环境、住宅、酒店、购物和市政设施，加快基础设施建设，完善各类公共服务，努力创造舒适便利的工作和生活环境。

二是打造优质高效的政务环境。继续推进优化发展环境专项整治，扎实开展第六批行政审批事项清理工作，规范审批程序，提高办事效率，打造审批环节最少、程序最简、办理最快、费用最低、服务最优的政务环境，推动政府职能向创造良好市场环境、提供优质公共服务、维护社会公平正义转变。完善“客商评价科股长”机制，突出整治行政机关庸懒散等不良风气，从严问责不作为、胡作为和乱作为，严厉打击强买强卖、强揽工程、恶意阻工等违法行为。

三是营造诚实守信的人文环境。强化“人人都是发展环境、人人都是商洛形象”的观念，切实加强干部群众的素质教育，提高广大群众的综合素质和文明素养。把诚信建设摆在突出位置，大力推进政务诚信、商务诚信、社会诚信和司法诚信建设。加强社会公德、职业道德、家庭美德和个人品德教育，充分发挥制度强制、行政导向、舆论监督等力量，努力在全社会形成良好的道德风尚。

(八)牢固树立以人为本，深入推进民生幸福工程

落实好“两个80%”的硬指标，深入推进各项利民惠民工作，不断满足人民群众对美好生活的新期待。

一是把增加群众收入作为最大实事。大力促进城乡居民就业增收，规范公务员津补贴和事业单位绩效工资制度，稳步提高企业退休人员养老金和企业最低工资标准，逐步提高工资性收入。加大强农惠农富农政策力度，继续增加对农民的直接补贴，稳步提高城乡居民医保财政补助标准，有效增加转移性收入。继续实施粮食高产创建、果业提质增效、畜牧收入倍增等农民增收工程，积极培植农产品加工和休闲农业等二三产业，扩大“三农”政策性保险范围，努力提高经营性收入。加快农村土地经营权和林权流转，引导农民进入农业园区就业，实现一份土地资源带来投资和工资两份收入，多渠道增加财产性收入。

二是把强化民生保障作为基本目标。加大民生项目建设力度，实施基本建设类民生项目1100个。实施更加积极的就业政策，积极开发公益性岗位，加强职业技能培训，做好困难群体就业援助。加快农民工返乡创业示范县建设，规范人力资源市场建设，以促进创业带动就业，鼓励多渠道多形式就业，切实做好以高校毕业生为重点的青年就业和农村转移劳动力、城镇困难人员、退役军人就业工作，全年新增城镇就业1.2万人，转移农村劳动力58.5万人。积极推进移民搬迁工程，坚持80%以上的集中安置率，提高上楼居民补助标准，新建集中安置点180个，全年搬迁1.6万户、6万人。继续加快保障性住房建设，完善住房分配、物业管理及有序退出等机制，新建保障性住房7100套，发放住房租赁补贴2500户。进一步完善覆盖城乡的社会保障体系，探索建立企业职工“五保合一”的统一征缴机制，增强社保基金筹资能力。解决好失独家庭养老问题。实行烈士子女生活补助。高度重视老龄和残疾人事业。全力保障妇女儿童合法权益，有效解决留守儿童、残疾流浪儿童的救助和教育问题。

三是把完善公共服务作为重要任务。继续实施学前一年免费教育，新建、改扩建公办幼儿园31所，力争市幼儿园建成投入使用，形成学前教育县区、镇、村三级服务网络体系。大力推进义务教育均衡发展，做好“双高双普”和教育强县创建。加强师资队伍建设，全面推进素质教育，支持发展高等教育，加快发展职业教育，着力促进教育公平，确保进城务工人员和进城落户农村居民子女平等接受教育。扎实推进农村义务教育“营养改善计划”规范实施。加快完善覆盖城乡居民的基本医药卫生制度，进一步提高城镇职工、居民基本医疗保险和新农合住院费用报销比例。积极推进城镇居民大病医疗救助试点，实施儿童白血病、先天性心脏病、终末期肾病等大病医疗保障，完成市、县区疾病预防控制、精神卫生、职业病和地方病防治等服务体系建设任务。深入实施文化惠民工程，不断健全基本公共文化体系。认真落实扶持文化产业发展的政策措施，重点培育发展以商洛花鼓、渔鼓为主的演出市场，以休闲为主的娱乐市场，以文物景点为主的旅游市场。开展“扫黄打非”，净化文化市场。加快改善体育基础设施，完成市体育场建设任务，举办好第三届全市运动会，促进全民健身活动广泛开展。坚持计划生育基本国策，继续稳定低生育水平，着力提高出生人口素质。

(九)加强和创新社会管理，提高社会管理科学化水平

强化政府社会管理和公共服务职能，加快构建新型社会管理服务体系，为经济社会发展创造和谐安定有序的社会环境。

一是加快推进城乡社区建设。探索建立新型社区管理体制，全面加快城乡社区服务站建设。继续鼓励高校毕业生面向农村基层就业，实行在优秀村干部中招收镇办公务员制度。以推进居住证为重点深化户籍制度改革，认真做好流动人口、农村留守人口和特殊人群的服务管理工作，努力使流动人口与居住地居民同享基本公共服务。

二是加强和改进信访工作。坚持用群众工作统揽信访工作，继续深化“三问三解”活动，健全信访工作责任制和矛盾纠纷排查调处制度，及时把群众反映强烈的问题解决在基层、化解在萌芽状态。加强“六五”普法和法律援助，完善司法基层基础工作。重视舆情研判、引导和处置，积极预防和妥善处置各类突发性事件。

三是持之以恒抓好安全生产。加强公共安全体系和应急能力建设，严格落实安全生产责任制，健全重大危险源分级监控机制，及时消除各类安全隐患，坚决防范各类安全事故发生。积极做好省级食品安全放心县区创建工作，突出抓好食品药品、医疗器械和农资等重要生活生产用品的质量安全专项整治，推进餐饮企业动态量化管理制度全覆盖。继续抓好学校食堂和校车安全管理。加强地质灾害防治，做好防灾减灾工作。

四是深化平安商洛创建活动。启动公安“三所”建设，健全社会治安防控体系，依法打击各类违法犯罪活动，进一步增强人民群众的安全感和满意度。强化国家安全和保密工作。全面落实民族、宗教和侨务政策，加快改善少数民族集中居住区条件，切实维护和保障各界群众的合法权益。加快推进人防工程建设。继续支持驻商部队正规化建设，完成市消防特勤中队营房建设，加快推进民兵训练基地和武警现代化建设，提高部队遂行多样化任务能力。

(十)加强政府自身建设，提高人民群众对政府的满意率

坚持以人为本、执政为民，加快转变政府职能，增强推动科学发展能力，努力建设人民满意的政府。

一是强化学习意识，开拓创新、增强能力。树立终身学习理念，争当勤学善思的表率。认真学习马克思主义、毛泽东思想、中国特色社会主义理论体系特别是科学发展观，学习党的各项路线、方针、政策和决议，学习推动事业发展所需的新观点新知识，在学习中解放思想、更新观念，在学习中增强本领、提升能力，在学习中发现机遇、抓住机遇，以开拓的思维、创新的举措，不断开创各项工作新局面。

二是强化法制意识，按规办事、依法行政。积极推进法治政府建设，继续做好依法行政示范市创建工作。自觉接受人大法律监督、政协民主监督，广泛听取各民主党派和人民团体的意见，虚心接受社会公众监督和新闻舆论监督。坚持市政府领导集体学法制度，做到政府职权法授、程序法定、行为法限、责任法究，不断提高运用法治思维和法治方法深化改革、推动发展、化解矛盾、维护稳定的能力。

三是强化实干意识，改进作风、狠抓落实。坚决落实中央“八项规定”及省委《实施意见》和市委《实施办法》，大力弘扬求真务实、苦干实干的工作作风。切实改进调查研究，切忌走过场、搞形式。切实改进文风、会风，精简会议、文件和简报，开短会、讲短话、求实效，严格控制和规范庆典、表彰、论坛、检查等活动。加大政府抓落实工作力度，严格绩效考核和行政问责，确保政令畅通、执行有力。

四是强化廉洁意识，清廉自律、树立形象。认真落实党风廉政建设责任制，全面推进惩治和预防腐败体系建设，严肃查处各类违法违纪行为，坚决纠正损害群众利益的不正之风。加大审计监督和效能监察力度，加强重点项目稽查监管和财政投资评审，规范政府采购和招投标行为，加强商业预付卡管理、“小金库”清理和公务卡结算工作。坚持勤俭节约办事，坚决反对铺张浪费，严格控制一般性支出。

各位代表，做好今年的工作，任务艰巨，责任重大。让我们紧密团结在以习近平同志为总书记的党中央周围，高举中国特色社会主义伟大旗帜，在省委、省政府和市委的坚强领导下，全面贯彻落实党的十八大精神，坚定信心，迎难而上，开拓进取，扎实工作，努力推动全市经济社会持续健康发展，为全面建设幸福商洛做出新的更大的贡献！
